# Validation and Comparison of Six Risk Scores for Infection in Patients With ST-Segment Elevation Myocardial Infarction Undergoing Percutaneous Coronary Intervention

**DOI:** 10.3389/fcvm.2020.621002

**Published:** 2021-01-22

**Authors:** Yuanhui Liu, Litao Wang, Wei Chen, Lihuan Zeng, Hualin Fan, Chongyang Duan, Yining Dai, Jiyan Chen, Ling Xue, Pengcheng He, Ning Tan

**Affiliations:** ^1^Guangdong Provincial Key Laboratory of Coronary Heart Disease Prevention, Department of Cardiology, Guangdong Cardiovascular Institute, Guangdong Provincial People's Hospital, Guangdong Academy of Medical Sciences, Guangzhou, China; ^2^School of Medicine, Guangdong Provincial People's Hospital, South China University of Technology, Guangzhou, China; ^3^The Second School of Clinical Medicine, Southern Medical University, Guangzhou, China; ^4^Fujian Provincial Key Laboratory of Cardiovascular Disease, Department of Cardiology, Fujian Provincial Center for Geriatrics, Fujian Cardiovascular Institute, Fujian Provincial Hospital, Provincial Clinical Medicine College of Fujian Medical University, Fuzhou, China; ^5^Department of Biostatistics, School of Public Health, Southern Medical University, Guangzhou, China

**Keywords:** risk score, infection, ST-segment elevation myocardial infarction, percutaneous coronary intervention, major adverse clinical events

## Abstract

**Aims:** Very few of the risk scores to predict infection in ST-segment elevation myocardial infarction (STEMI) patients undergoing percutaneous coronary intervention (PCI) have been validated, and reports on their differences. We aimed to validate and compare the discriminatory value of different risk scores for infection.

**Methods:** A total of 2,260 eligible patients with STEMI undergoing PCI from January 2010 to May 2018 were enrolled. Six risk scores were investigated: age, serum creatinine, or glomerular filtration rate, and ejection fraction (ACEF or AGEF) score; Canada Acute Coronary Syndrome (CACS) risk score; CHADS_2_ score; Global Registry for Acute Coronary Events (GRACE) score; and Mehran score conceived for contrast induced nephropathy. The primary endpoint was infection during hospitalization.

**Results:** Except CHADS_2_ score (AUC, 0.682; 95%CI, 0.652–0.712), the other risk scores showed good discrimination for predicting infection. All risk scores but CACS risk score (calibration slope, 0.77; 95%CI, 0.18–1.35) showed best calibration for infection. The risks scores also showed good discrimination for in-hospital major adverse clinical events (MACE) (AUC range, 0.700–0.786), except for CHADS_2_ score. All six risk scores showed best calibration for in-hospital MACE. Subgroup analysis demonstrated similar results.

**Conclusions:** The ACEF, AGEF, CACS, GRACE, and Mehran scores showed a good discrimination and calibration for predicting infection and MACE.

## Introduction

Infection complicating the course of ST-segment elevation myocardial infarction (STEMI) is uncommon, with a reported incidence of 2.4%; nonetheless, it is associated with markedly worse 90-day clinical outcomes and longer hospital stay (1). Since a substantial proportion of infections are considered preventable, identifying patients at risk of infection is essential to estimate patients' prognosis, aid in clinical decision making, and ensure quality control. However, current data on infection prediction in patients with STEMI undergoing percutaneous coronary intervention (PCI) are limited.

Some commonly used risk scores in clinical practice, such as the age, serum Creatinine (sCr), or Glomerular filtration rate, and Ejection Fraction (ACEF or AGEF) score, Canada Acute Coronary Syndrome (CACS) score, CHADS_2_ score, Global Registry for Acute Coronary Events (GRACE) score and Mehran score (conceived for contrast induced nephropathy) have been reported to predict several clinical outcomes in patients with STEMI who have undergone PCI (2–7), and all scores include some risk factors of infection (age, heart failure, diabetes mellitus and so on). Additionally, our recent studies showed that the ACEF/AGEF, and CACS scores performed well in predicting infection in STEMI patients (8, 9). However, the use of other risk scores for infection prediction in such patients has not been fully examined and validated in large-sample studies. Thus, the present study aimed to validate and compare the performance of six risk scores for infection in patients with STEMI undergoing PCI.

## Methods

### Study Population

This observational cohort study prospectively enrolled a series of consecutive patients with STEMI undergoing PCI after symptoms onset in the department of cardiac care unit at Guangdong Provincial People's Hospital from January 2010 to May 2018. STEMI was defined as the manifestation of typical chest pain and concomitant symptoms for ≥ 30 min but <12 h, with ST-segment elevation ≥ 1 mm in ≥ 2 continuous leads or new or undetermined duration of left branch bundle block accompanying with ≥ 2 times increase in troponin I or T (10). Patients diagnosed with infection prior to admission were excluded. Patients with any of the following conditions were also excluded from the study: (a) tumors or chronic inflammatory diseases, (b) on hemodialysis at admission for chronic renal failure, (c) undergoing emergency cardiac surgery, (d) died within 24 h after admission, (e) readmission to hospital and (f) with missing variables that are needed to calculate the risk scores. The study protocol was approved by the ethics and research committee of our hospital and performed in accordance with the principles of the Declaration of Helsinki. Informed consent was obtained from the patients prior to study participation. This study was registered with the Chinese Clinical Trial Registry (ChiCTR 1900028278).

### Procedure and Medications

Baseline demographic characteristics and relevant clinical information required for calculating the different scores of each patient were obtained from the hospital electronic database. All included patients had a routine chest X-ray, and ultrasonic cardiography was conducted after admission. Blood, urine, and sputum cultures were examined at the discretion of the physicians. All patients were treated in accordance with our institution's protocol and the European Society of Cardiology guidelines (10). A 24 h on-call interventional team performed PCI, following standard clinical practice and techniques. All eligible patients were administered aspirin (300 mg), and clopidogrel (300 or 600 mg) or ticagrelor (180 mg) before PCI. After the PCI procedure, the patients received dual antiplatelet therapy (aspirin, 100 mg/day combined with clopidogrel, 75 mg/day, or ticagrelor, 90 mg twice/day). According to clinical protocols that are based on interventional guidelines (10), prescription of anticoagulants, glycoprotein IIb/IIIa inhibitors, β blockers, or angiotensin-converting enzyme inhibitors/angiotensin receptor blockers was at the cardiologist's discretion.

### Score Calculations

Score calculations were performed by experienced cardiologists who were blinded to the outcomes. The ACEF and AGEF scores were calculated within 24 h after admission using the following formula: ACEF = age/left ventricular ejected fraction (LVEF) +1 (if the sCr level was > 2 mg/dL) (11), and AGEF = age/LVEF (%) +1 (if the estimated glomerular filtration rate (eGFR) was < 60 mL/min/1.73 m^2^) (3). In addition, the eGFR was calculated using the 4-variable modification of diet in renal disease equation (12). The CACS score was calculated on a scale of 0–4 at admission, based on four clinical parameters (1 point for each): age ≥ 75 years, Killip class > 1, systolic blood pressure < 100 mmHg, and heart rate > 100 beats/min) (4). For CHADS_2_ score, 2 points were assigned for history of stroke or transient ischemic attack and 1 point for congestive heart failure, hypertension, age ≥ 75 years, and diabetes mellitus (5). The GRACE score, which included eight variables (i.e., age, heart rate, systolic blood pressure, creatinine, Killip's classification, cardiac arrest at admission, increased cardiac markers and ST-segment deviation), was calculated according to the computational method (Supplementary Table 1) (6). Mehran score, which included the following eight variables: hypotension, congestive heart failure, chronic kidney disease, age > 75 years, anemia, diabetes, contrast medium volume, and use of intra-aortic balloon pump, was defined according to the specifications of Mehran et al. (13). In order to compare the differences among risk scores, patients were assigned into categories according to tertiles from the present data. Patients in tertiles I, II, and III of each risk score were considered as low-risk, moderate-risk, and high-risk populations, respectively.

### Study Endpoints

The primary endpoint was infection during hospitalization, which was diagnosed based on signs, symptoms and laboratory tests compatible with infection. In addition, infection was categorized as pulmonary infections, urinary infections or others (i.e., primary bacteremia, abdominal sepsis, and unidentified primary infection site), according to the clinical records during hospitalization. Appropriate antibiotics were administered once infection was confirmed (14). The hospital infection control committee validated the antibiotics and approved their initiation for each infection case according to the hospital's infection prevention and control regime. The secondary endpoints were in-hospital MACE, which was defined as a composite endpoint of all-cause death, recurrent myocardial infarction, target vessel revascularization, stroke, and renal replacement therapy during hospitalization.

### Statistical Analysis

Chi-squared test or Fisher's exact test was used, as appropriate, to compare the categorical variables, which were expressed as percentages. Comparisons between normally distributed continuous variables, which were expressed as mean ± SD, were performed using two-sample *t-*tests. Non-normally distributed continuous variables, presented as median and interquartile range, were analyzed using Wilcoxon rank sum tests. Area under the receiver-operating characteristic curves (AUC) was constructed to assess the discrimination of risk scores for infection and MACE, which was expressed by c-statistic. An AUC < 0.60 was considered to reflect poor discrimination, 0.60–0.75 good discrimination, and > 0.75 best discrimination (15). AUCs were compared using the non-parametric approach of DeLong et al. (16). An objective assessment of calibration was analyzed using the Hosmer-Lemeshow goodness-of-fit test (17); calibration slope (plotting the observed proportions vs. the predicted probabilities), with 1 indicated perfect calibration. In addition, decision curve analysis was introduced to evaluate the utility of the risk scores; the risk scores with a higher net benefit across the range of thresholds indicated better clinical usefulness (18). All tests were two tailed, and a *P* < 0.05 was considered statistically significant. All data analyses were performed using SAS version 9.4 (SAS Institute, Cary, NC).

## Results

### Baseline Clinical Characteristics

**Figure 1:**
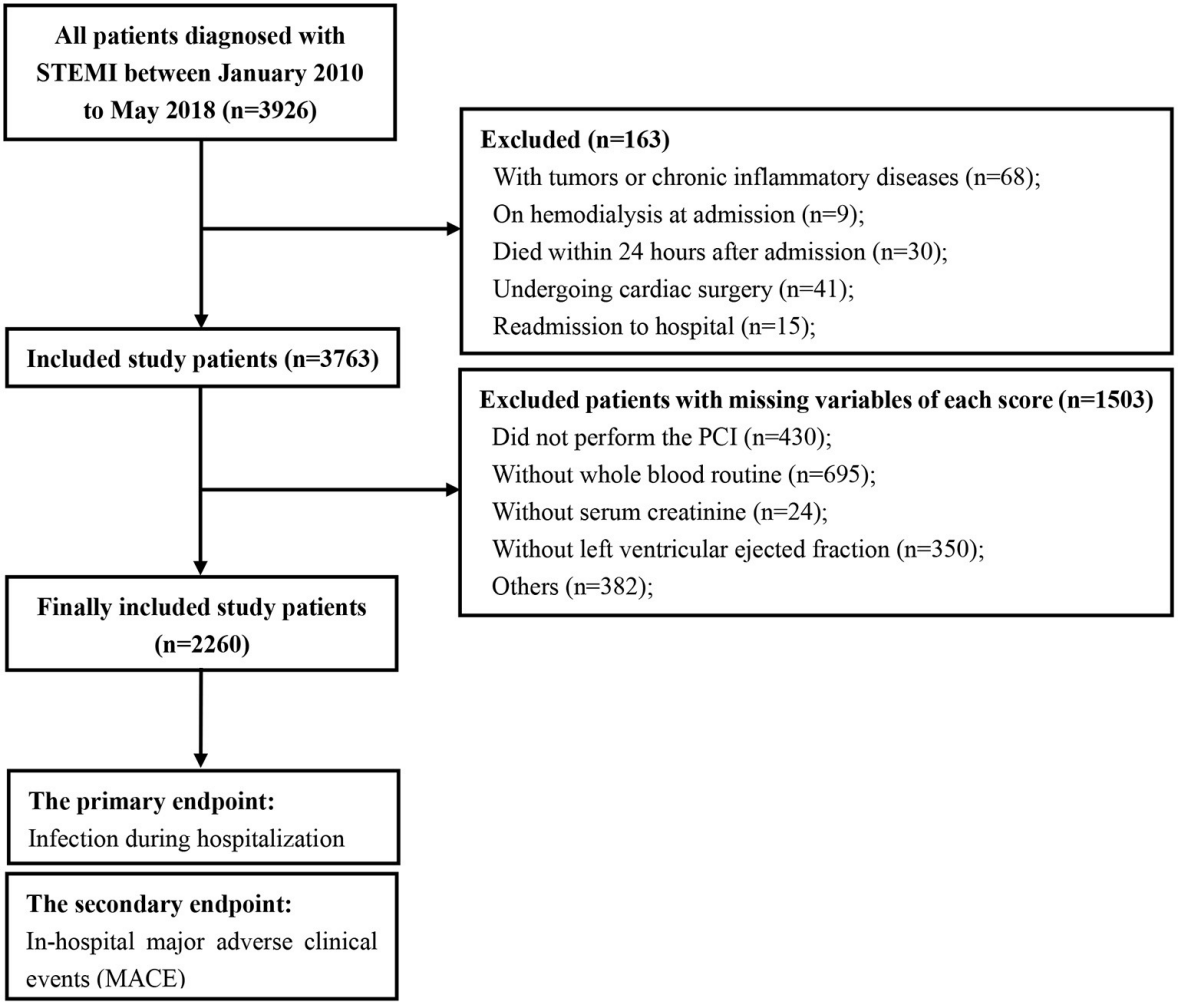
Study flow of participants. STEMI, ST-segment elevation myocardial infarction; PCI, percutaneous coronary intervention.

The study flow is shown in Figure 1. A total of 2,260 consecutive patients with STEMI (62.32 ± 12.36 years; 81.3% males) were finally included. All patients' baseline clinical characteristics and the mean of all risk scores are presented in Table 1. Briefly, 51.3% patients had hypertension, 28.4% had diabetes, and 30.5% had prior myocardial infraction. Mean eGFR, LVEF, and contrast volume were 80.87 ± 29.53 mL/min/1.73 m^2^, 51.60 ± 11.77% and 112.56 ± 45.12 mL, respectively. The overall incidence of infection during hospitalization was 16.0%, the incidence of pulmonary infections and urinary infections was 70.6 and 15.0%, respectively. The rate of mechanical ventilation was 6.2% during hospitalization, and the rate of in-hospital MACE was 7.6% (Table 1).

**Table 1:** Baseline characteristics of the study population.

**Variables**	
Age (years)	62.32 ± 12.36
Age > 75 year, n (%)	1,004 (44.4%)
Male sex, n (%)	1,838 (81.3%)
Systolic blood pressure, (mmHg)	122.36 ± 21.34
Diastolic blood pressure, (mmHg)	74.10 ± 13.18
Heart rate (bpm)	79.53 ± 15.55
**Killip class**	
I, n (%)	1,588 (70.3%)
II, n (%)	482 (21.3%)
III, n (%)	115 (5.1%)
IV, n (%)	75 (3.3%)
Smoke, n (%)	890 (39.4%)
**Medical history, n (%)**	
Hypertension	1,158 (51.2%)
Diabetes	642 (28.4%)
Prior myocardial infraction	690 (30.5%)
Coronary artery bypass graft	4 (0.2%)
COPD	53 (2.3%)
Atrial fibrillation	76 (3.4%)
Stroke	153 (6.8%)
Anemia	690 (30.5%)
Chronic heart failure	672 (29.7%)
Mechanical ventilation	140 (6.2%)
**Laboratory characteristics**	
eGFR, (mL/min/1.73m^2^)	80.87 ± 29.53
Serum creatinine, (mg/dL)	1.19 ± 0.92
Serum albumin, (g/L)	34.86 ± 4.32
White blood cell, 10^9^/L	11.41 ± 3.95
Hemoglobin, (g/L)	134.63 ± 18.70
Triglyceride, (mmol/L)	1.62 ± 1.09
Total cholesterol, (mmol/L)	4.84 ± 1.26
Left ventricular ejection fraction, (%)	51.60 ± 11.77
High-sensitivity C-reactive protein, (mg/L)	3.09 (1.3–7.5)
**Medication during hospital stay, n (%)**	
Glycoprotein IIb/IIIa inhibitors	823 (36.5%)
Statins	2,202 (97.5%)
Warfarin	33 (1.5%)
ACEI	1,373 (60.9%)
Calcium channel blockers	190 (8.4%)
Angiotensin receptor blockers	310 (13.7%)
β-blockers	1,854 (82.1%)
**Procedural characteristics**	
Radial access, n (%)	1,936 (85.7%)
Femoral assess, n (%)	322 (14.3%)
Multi-lesion, n (%)	1,837 (81.3%)
Contrast volume, (mL)	112.56 ± 45.12
Contrast volume ≥ 100 mL, n (%)	1,642 (72.7%)
Number of stents, (n)	1.39 ± 2.38
Total length of stent, (mm)	34.37 ± 24.37
**Risk scores (Low, Moderate, High)**	
ACEF score	1.35±0.60 (≤1.05, 1.05–1.41, >1.41)
AGEF score	1.51±0.75 (≤1.07, 1.07–1.58, >1.58)
CACS score	0.71 ± 0.84 (0, 0–1, >1)
CHADS_2_ score	1.41 ± 1.22 (≤1, 1–2, >2)
GRACE score	160.99 ± 33.68 (≤144, 144–172, >172)
Mehran score	8.77 ± 6.18 (≤6, 6–10, >10)
**In hospital events**	
In-hospital day, median (IQR)	6 (5–9)
All-cause death, n (%)	104 (4.6%)
In-hospital MACE	172 (7.6%)

*Values are mean ± SD, n (%) or median (interquartile range)*.

*COPD, chronic obstructive pulmonary disease; eGFR, estimated glomerular filtration rate; ACEI, Angiotensin converting enzyme Inhibitors; ACEF, age, creatinine, and ejection fraction; AGEF, age, glomerular filtration rate, and ejection fraction; CACS, Canada acute coronary syndrome; GRACE, global registry for acute coronary events; IQR, interquartile range; MACE, major adverse clinical events*.

**Figure 2:**
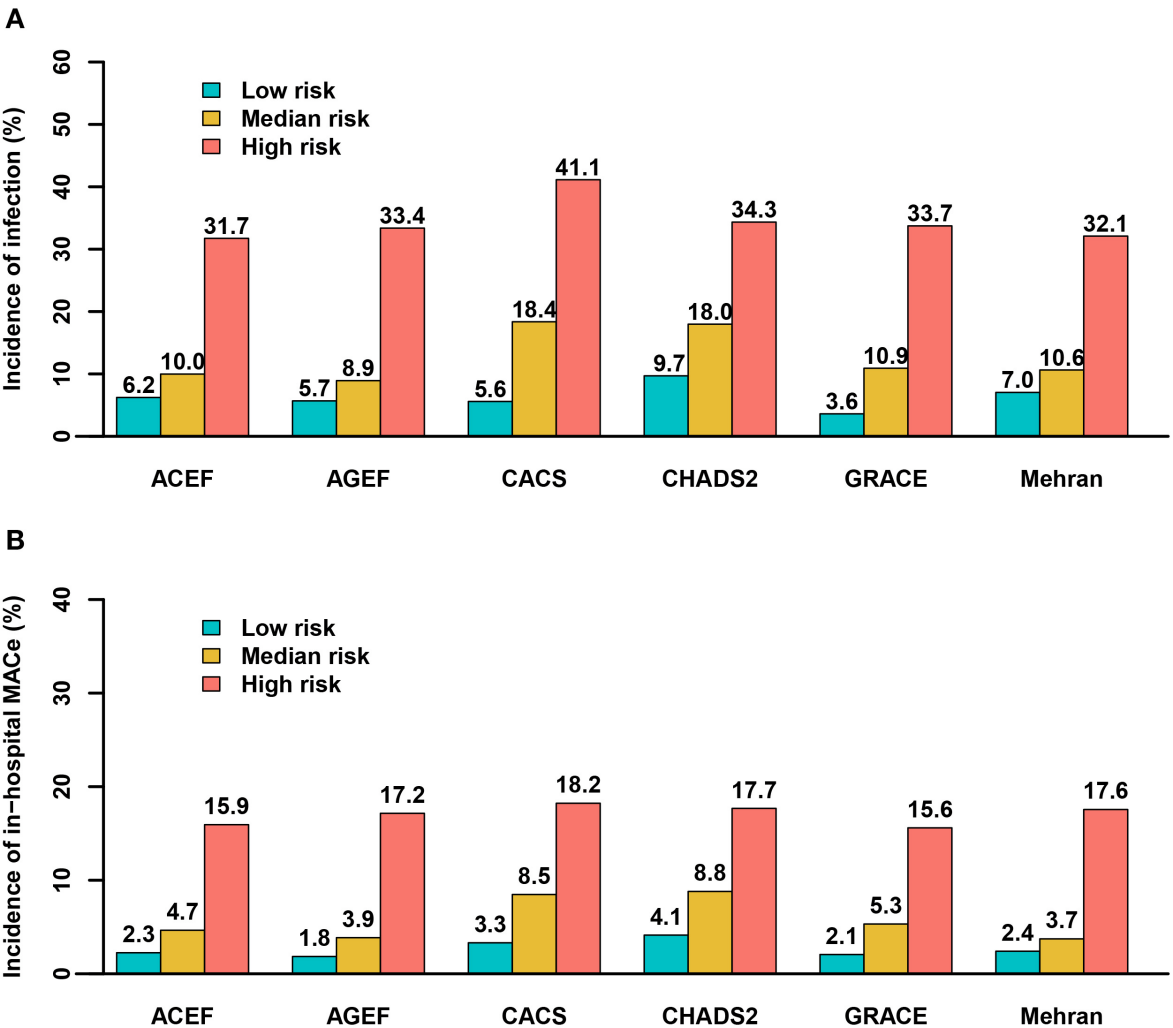
Predictive ability of the risk scores for infection and major adverse clinical events by tertiles. **(A)** infection; **(B)** MACE. ACEF (≤1.05, 1.05–1.41, >1.41); AGEF (≤1.07, 1.07–1.58, >1.58); CACS (0, 0–1, >1); CHADS2 (≤1, 1–2, >2); GRACE (≤144, 144–172, >172); Mehran (≤6, 6–10, >10).

All six risk scores showed abnormal distribution (*P* < 0.01) in Supplementary Figure 1. Clinical outcomes according to tertiles (low-, moderate-, and high-risk groups) of all the six risk scores are shown in Figure 2. A significant positive gradient of risk with respect to infection and MACE was observed as the risk scores increased.

### Discrimination for Infection and In-hospital MACE

The prognostic accuracy of the six risk scores for infection and in-hospital MACE are presented in Table 2 and Supplementary Figure 2. Each score showed a clear discrimination for infection, with AUCs ranging from 0.746 to 0.791, except for CHADS_2_ risk score [AUC, 0.682; 95% confidence interval [CI], 0.652–0.712] (Table 2 and Supplementary Figure 2). Additionally, the prognostic accuracy of all risk scores was assessed according to the subtypes of infection. CHADS_2_ score had a relatively poor predictive value for pulmonary infection (AUC, 0.675; 95% CI, 0.639–0.710). CHADS_2_ and Mehran risk score showed poor predictive value for urinary infection (AUC, 0.653 for CHADS_2_; AUC, 0.697 for Mehran, respectively), and other risk scores displayed good predictive power for both pulmonary infection and urinary infection (AUC > 0.70).

**Table 2:** Predictive accuracy of the risk scores for infection and major adverse clinical events.

**Events**	**ACEF**	**AGEF**	**CACS**	**CHADS_**2**_**	**GRACE**	**Mehran**
	**AUC (95%CI) P1/P2**					
**Infection**	0.748 (0.720–0.777)	0.770 (0.742–0.798)	0.746 (0.720–0.772)	0.682 (0.652–0.712)	0.791 (0.765–0.817)	0.752 (0.723–0.782)
			0.885/0.132	<0.001/ <0.001	0.002/0.100	0.800/0.226
**MACE**	0.771 (0.732–0.809)	0.786 (0.749–0.823)	0.700 (0.660–0.740)	0.696 (0.657–0.735)	0.761 (0.722–0.799)	0.779 (0.739–0.819)
			0.003/ <0.001	0.001/ <0.001	0.616/0.171	0.700/0.717

*P1: other risk scores (but not AGEF) vs. ACEF; P2: other risk scores (but not ACEF) vs. AGEF*.

*MACE, major adverse clinical events; ACEF, age, creatinine, and ejection fraction; AGEF, age, glomerular filtration rate, and ejection fraction; CACS, Canada acute coronary syndrome; GRACE, global registry for acute coronary events; AUC, area under the curve; CI, confidence interval*.

Furthermore, each score had a best discrimination for in-hospital MACE, with AUCs ranging from 0.700 to 0.786, except for CHADS_2_ risk score, with AUC of 0.696 (Table 2 and Supplementary Figure 2).

### Predictive Values of Risk Scores for Subgroup Analysis

Subgroup analysis was performed according to the WBC count (≥10 × 10^9^/L or <10 × 10^9^/L), gender and hypertension. The results showed similar discrimination of these 6 risk scores for infections in these subgroups with the primary analysis (Supplementary Tables 2–4). However, the discriminative ability of these 6 risk scores for infection was relative lower for female than those for male (Supplementary Table 3).

### Calibration of Risk Scores for Infection and In-hospital MACE

**Figure 3:**
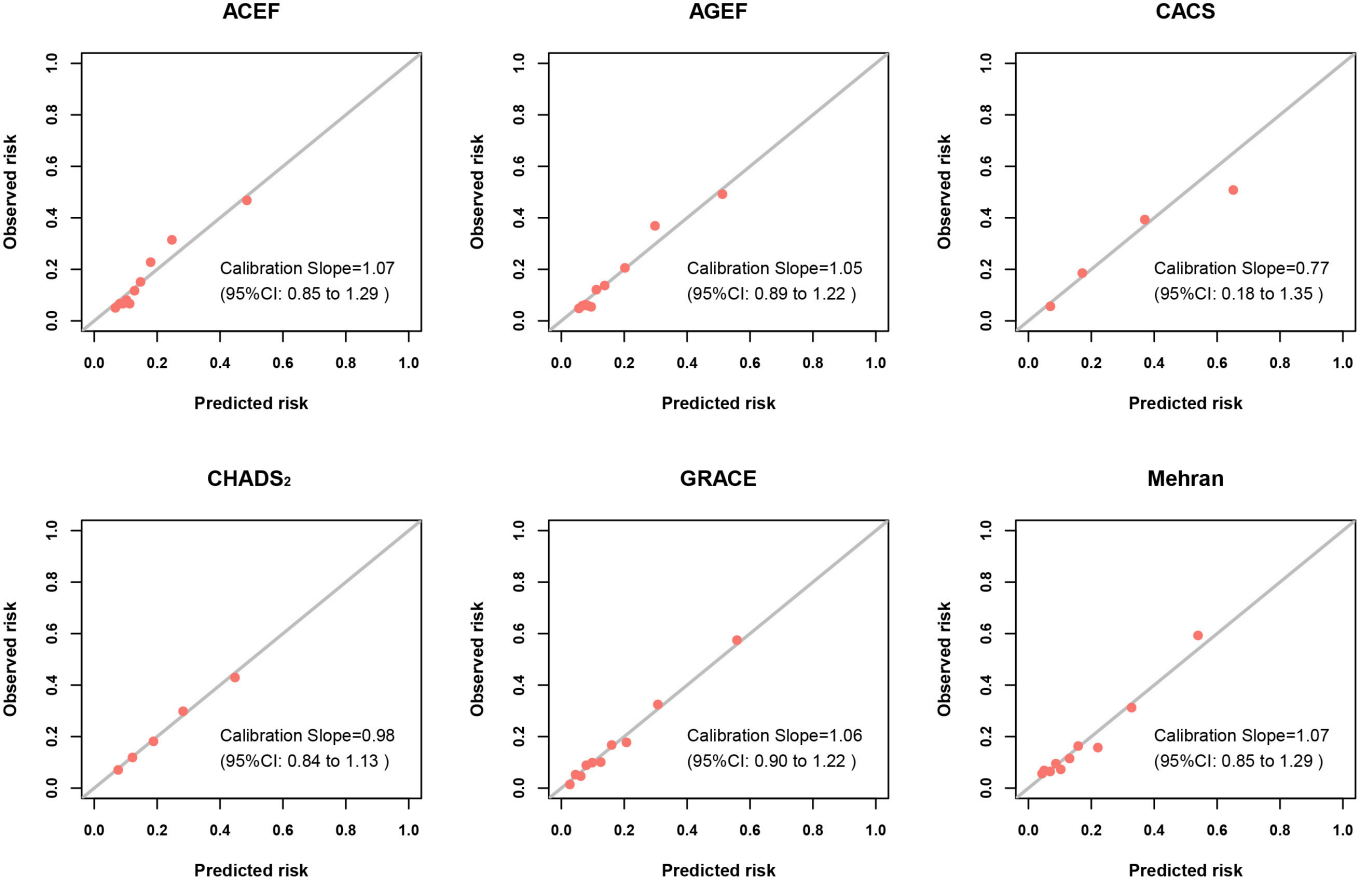
Calibration plots of risk scores for infection. Calibration plots showing the predicted probability vs. observed incidence of infection of risk scores. The diagonal line represents the perfect calibration (curve with a slope of 1 and an intercept of 0).

Figure 3 and Supplementary Figure 3 illustrated the calibration plots of the six risk scores for infection and MACE during hospitalization. All risk scores exhibited best calibration for in-hospital infection (calibration slope nearly at 1), while CACS risk score displayed good calibration (calibration slope, 0.77; 95%CI 0.18–1.35) (Figure 3). Moreover, for in-hospital MACE, the best calibration was found in all risk scores (calibration slope nearly at 1) (Supplementary Figure 3).

### Utility of Risk Scores

When used to predict infection during hospitalization in patients with STEMI undergoing PCI, ACEF, AGEF, CACS, GRACE, and Mehran scores showed best discrimination (AUC ≥ 0.75) and good calibration abilities. And CHADS_2_ risk score had a relatively good calibration but low discrimination for infection. With regard to the prediction for in-hospital MACE, all risk scores showed best calibration abilities (calibration slope nearly at 1), and all risk scores except CHADS_2_ risk scores showed good discrimination (AUC ≥ 0.70).

**Figure 4:**
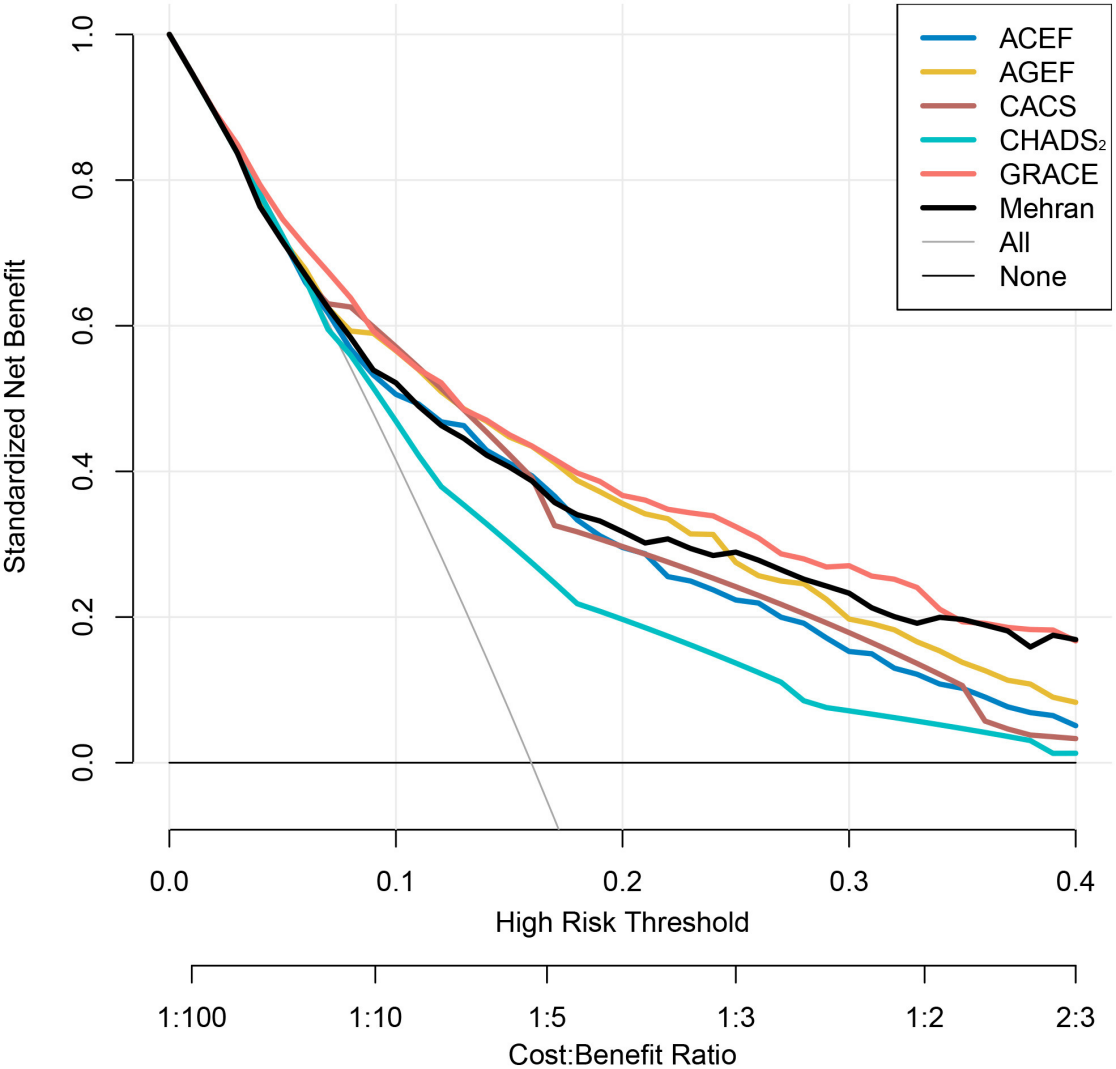
Decision curves of risk scores for infection. Net benefit of using models to predict infection development are shown. The GRACE risk score (Red) demonstrated the greatest standardized net benefit while CHADS2 risk score showed the lowest (Green).

In addition, the decision curves of the risk scores for infection and in-hospital MACE are shown in Figure 4 and Supplementary Figure 4, respectively. Except CHADS_2_ risk score, the other risk scores had a good standardized net benefit for infection; GRACE risk score had the greatest standardized net benefit. The risk scores, except CHADS_2_ and CACS risk score, exhibited a good standardized net benefit for in-hospital MACE.

## Discussion

To our knowledge, this study is the first to validate and compare the prognostic values of six risk scores for infection and in-hospital MACE in patients with STEMI undergoing PCI. The major findings are as follows: (1) the risk scores, except CHADS_2_ risk score, showed good discrimination and calibration for infection, and the GRACE risk score was slightly superior to the other risk scores; (2) all six risk scores showed good discrimination and calibration for in-hospital MACE; and (3) among the six risk scores, the CACS risk score was preferentially recommended for clinical use as its clinical variables were simpler and more practical.

Infection, which is an important complication of patients with STEMI following PCI, is associated with significantly worse clinical outcomes (1). A recent prospective study reported that in-hospital mortality was twice higher in patients with MI concurrent of infection than those patients without infection (19). In our study, 361 patients (16.0%) had infection, which was slightly higher than the incidences in the previous studies (3.9 and 10%) (14, 19). Several studies have reported that infection is a common problem for intensive care unit (ICU) patients, and the risk of infection increases with the duration of ICU stay (20); thus, the higher rate of infection in our study may be associated with prolonged ICU stay (6 days vs. 1 day) (21). In addition, about 30% patients in Killip class ≥ II, suggesting a complicated situation, that can prolong hospital stay and the patients can be more prone to infections. Hence, identifying patients with STEMI with a high risk of infection is crucial. However, data on infection risk assessment in such patients are scarce.

Scoring systems currently used for clinical event risk stratification in patients with STEMI are based on multivariable models that integrate elements from medical history, admission electrocardiogram, biochemical evidence of myocyte necrosis, and renal function (2–7). Previous reports have proposed criteria for predicting infection in cardiovascular diseases, and mostly, in cardiac surgery (22, 23). Before the ACEF, AGEF, and CACS risk scores were firstly validated in our recent studies, a prediction score for infection in patients with STEMI undergoing PCI had not been reported (8, 9). Although none of the six risk scores were developed specifically for predicting infection, this study found that they could predict infection well and thus their use was expanded. The variables included in these risk scores were associated with the risk of infection. Thus, the good predictive value for infection was somehow expected. Previous studies demonstrated that renal function and hemodynamic status, including heart rate, and blood pressure, are associated with the development of infection (24–26). In addition, the complex and vicious interaction between heart failure and infection has been reported in many studies (27, 28). An increase in pulmonary pressure, that is induced by the decline in LVEF, results in pulmonary edema and accumulation of pneumonia related pathogens, such as Streptococcus pneumoniae, which has a deleterious effect and could lead to respiratory infection (29).

The GRACE risk score, which is a useful scoring model to predict short-term mortality in acute MI, has a high diagnostic accuracy for the prediction of 6-month post-discharge death in patients with acute coronary syndrome treated with primary PCI (6). Among the six risk scores, the GRACE risk score provided the greatest discrimination for infection risk prediction. However, the GRACE risk score requires evaluation of eight variables, and should be calculated using a computer, thereby making its use in the clinical setting challenging; a simpler risk score is required for widespread adoption. Moreover, compared to the GRACE and Mehran risk scores, the ACEF, AGEF, and CACS risk scores, which include three to four variables, are less complicated and also had best discrimination and good calibration for infection. Furthermore, the decision curves showed that these three risk scores had a good standardized net benefit for infection. Nevertheless, the ACEF and AGEF risk scores include the heart function evaluated by echocardiography and renal function measured by serum creatinine, whereas, the CACS risk score included accessible clinical variables only in clinical practice, which are simple to remember, and calculate, and thus could be time-saving. The CHADS_2_ score is a useful prognostic tool for predicting cardiovascular or cerebrovascular events in patients with acute coronary syndrome (30). In our study, the CHADS_2_ score showed good calibration but a relatively low discrimination, and bad standardized net benefit for infection, which in turn is of limited clinical use. A potential explanation of such low discrimination is that unlike hypotension, hypertension in the CHADS_2_ risk score was not proven to be associated with the risk of infection (26), thereby reducing the predictive value of CHADS_2_ risk score to some extent.

Although the six risk scores could predict clinical outcomes in patients with STEMI, differences in their prediction of in-hospital MACE remained unclear. Our study is the first to investigate the difference in predicting MACE among the risk scores. Of the six risk scores, ACEF, AGEF, GRACE, and Mehran risk scores showed best discrimination, CACS risk score and CHADS_2_ risk score showed good discrimination, and all risk scores showed best calibration. Nevertheless, considering its discrimination, calibration, and clinical simplicity, the CACS risk score may be a good tool for predicting MACE.

Notably, recent study has demonstrated that gender played an active role in the incidence and outcomes of major infectious diseases, such as community-acquired pneumonia (31). However, another investigation reported an opposite result that gender did not appear to play a role in acquisition of an intensive care unit-acquired infection in critically ill patients (32). In current study, the discriminative ability of these 6 risk scores for infection was relative lower for female than those for male, which suggested that gender difference might alter the prognostic capacity to some extent. Furthermore, Ishigami et al. (33) revealed that patients with severe hypertension on admission appeared to be at increased risk of stroke-associated pneumonia in elderly subjects with acute ischemic stroke. The result indicated that hypertension might contribute to the infection development. However, we demonstrated that all six risk scores displayed similar fair discriminative capacity for infection in patients with or without hypertension. Thus, further investigations might be required to examine the effects of hypertension on the prediction of these risk scores.

Our study also had several limitations. First, this study was performed at a single center; thus, the results should be interpreted prudently. Second, the predictive values observed were exclusive to patients with STEMI; hence, caution should be taken in applying the findings to other patients. Lastly, we only included six risk scores to predict infection because we believe that these risk scores are commonly used and those not included are excessively complicated. Future research may consider examining the predictive values of other risk scores in different populations.

In summary, for patients with STEMI undergoing PCI, ACEF, AGEF, CACS, GRACE, and Mehran risk score have good discrimination and calibration for predicting infection and in-hospital MACE during hospitalization. Of the six risk scores investigated in this study, the CACS risk score is preferentially recommended for clinical use as its variables are simpler and practical.

## Data Availability Statement

The raw data supporting the conclusions of this article will be made available by the authors, without undue reservation.

## Ethics Statement

The studies involving human participants were reviewed and approved by Ethics and Research Committee of Guangdong Provincial People's Hospital. The patients/participants provided their written informed consent to participate in this study.

## Author Contributions

NT, PH, and YL: study design. LW, WC, LZ, HF, and YD: data collection. CD, YL, and LW: data analysis and interpretation. YL, LW, and WC: manuscript preparation. All authors: critical revision of manuscript.

## Conflict of Interest

The authors declare that the research was conducted in the absence of any commercial or financial relationships that could be construed as a potential conflict of interest.
