# Supplementary material for: Validation and Comparison of Six Risk Scores for Infection in Patients With ST-Segment Elevation Myocardial Infarction Undergoing Percutaneous Coronary Intervention
Source: Front Cardiovasc Med. 2021 Jan 22;7:621002. doi: 10.3389/fcvm.2020.621002 (PMC7862339; doi:10.3389/fcvm.2020.621002)
Supplement: Supplementary file 1 [file Data_Sheet_1.docx]

**Supplemental materials**

**Validation and comparison of six risk scores for infection in patients with** **ST-segment elevation myocardial infarction undergoing percutaneous coronary intervention**

Yuanhui Liu*, MD, PhD; Litao Wang*, MD; Wei Chen*, MD; Lihuan Zeng, MD; Hualin Fan, MD; Chongyang Duan, MD, PhD; Yining Dai, MD; Jiyan Chen, MD, PhD; Ling Xue, MD, PhD; Pengcheng He^#^, MD, PhD; Ning Tan^#^, MD, PhD.

**Contents**

**Figure legends**

**Figure S1.** Distributions of the risk scores for all patients.

A for ACEF score; B for AGEF score; C for CACS score; D for CHADS_2_ score; E for GRACE score; F for Mehran score.

**Figure S2.** Receiver operating characteristic curves of the risk scores for infection, major adverse clinical events, pulmonary infection and urinary infection.

A for infection; B for major adverse clinical events; C for pulmonary infection; D for urinary infection.

**Figure S3.** Calibration plots of risk scores for major adverse clinical events.

**Figure S4.** Decision curves of the risk scores for major adverse clinical events.

**Table legends**

**Table S1.** Variables in the risk scores evaluated.

**Table S2.** Predictive accuracy of the risk scores for infection in patients with different WBC count.

**Table S3.** Predictive accuracy of the risk scores for infection in male and female.

**Table S4.** Predictive accuracy of the risk scores for infection in patients with or without hypertension.

**Figure S1.** Distributions of the risk scores for all patients.


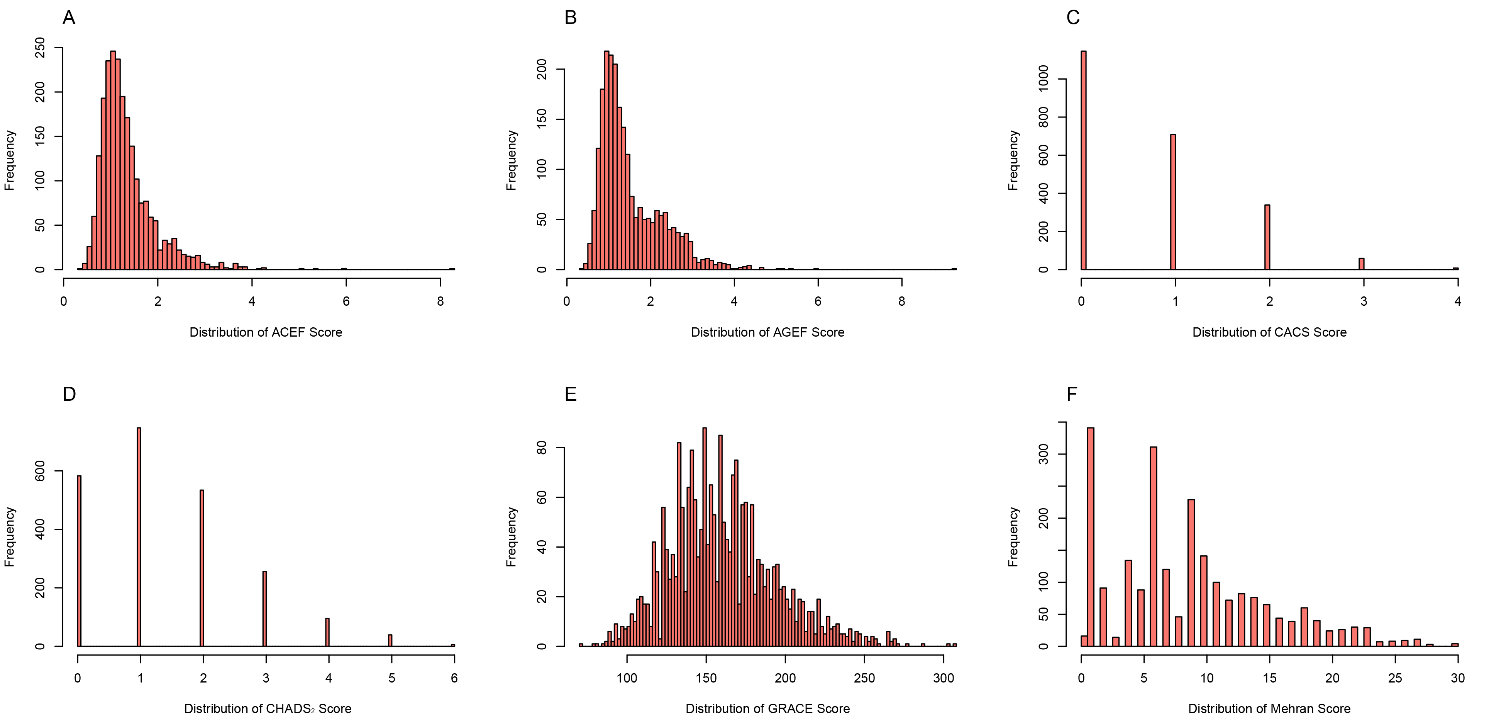


A for ACEF score; B for AGEF score; C for CACS score; D for CHADS_2_ score; E for GRACE score; F for Mehran score. *ACEF, age, creatinine, and ejection fraction; AGEF, age, glomerular filtration rate, and ejection fraction; CACS, Canada acute coronary syndrome; GRACE, global registry for acute coronary events.*

**Figure S2.** Receiver operating characteristic curves of the risk scores for infection, major adverse clinical events, pulmonary infection and urinary infection.


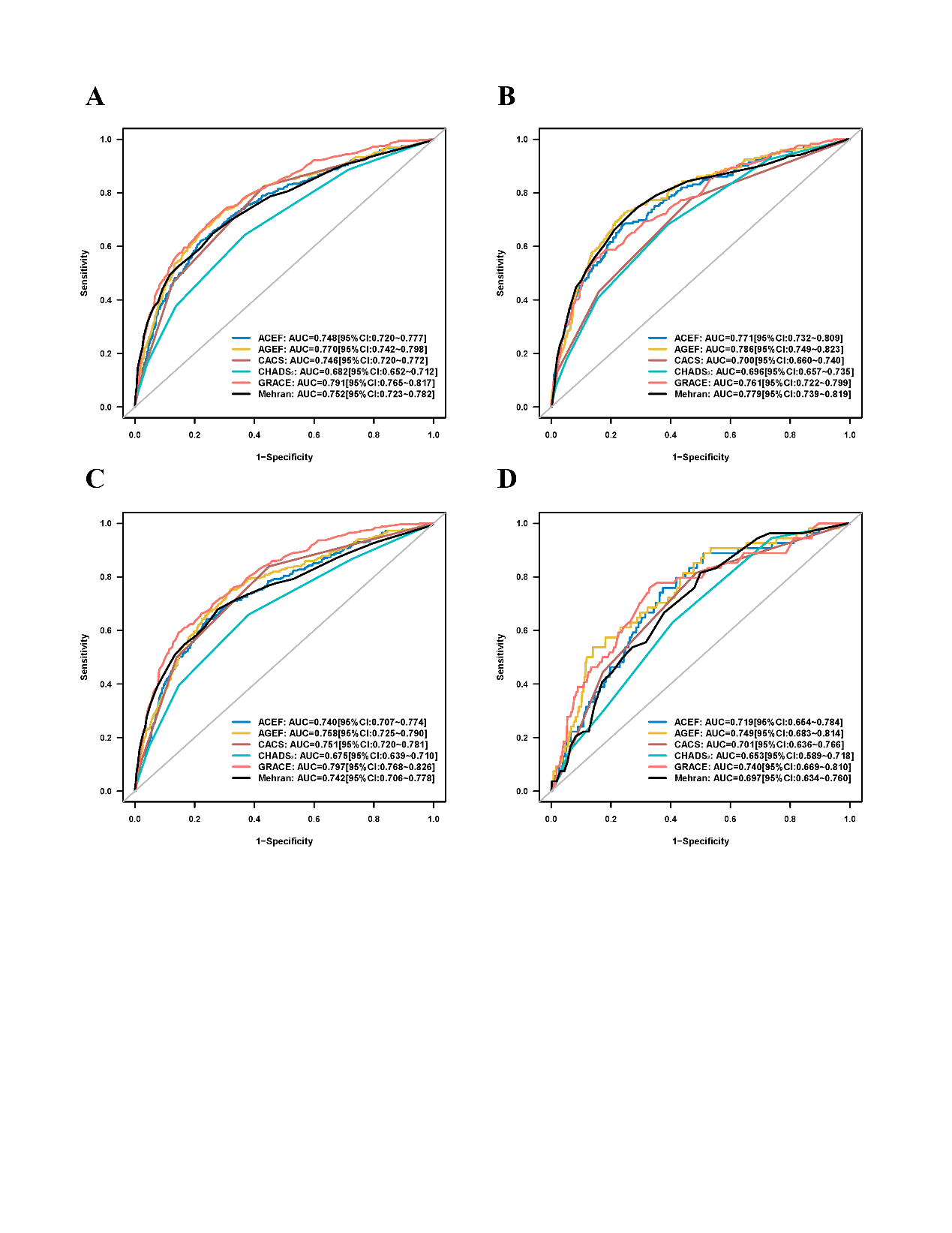


A for infection; B for major adverse clinical events; C for pulmonary infection; D for urinary infection.

*ACEF, age, creatinine, and ejection fraction; AGEF, age, glomerular filtration rate, and ejection fraction; CACS, Canada acute coronary syndrome; GRACE, global registry for acute coronary events; AUC, area under the curve; CI, confidence interval.*

**Figure S3.** Calibration plots of risk scores for major adverse clinical events.


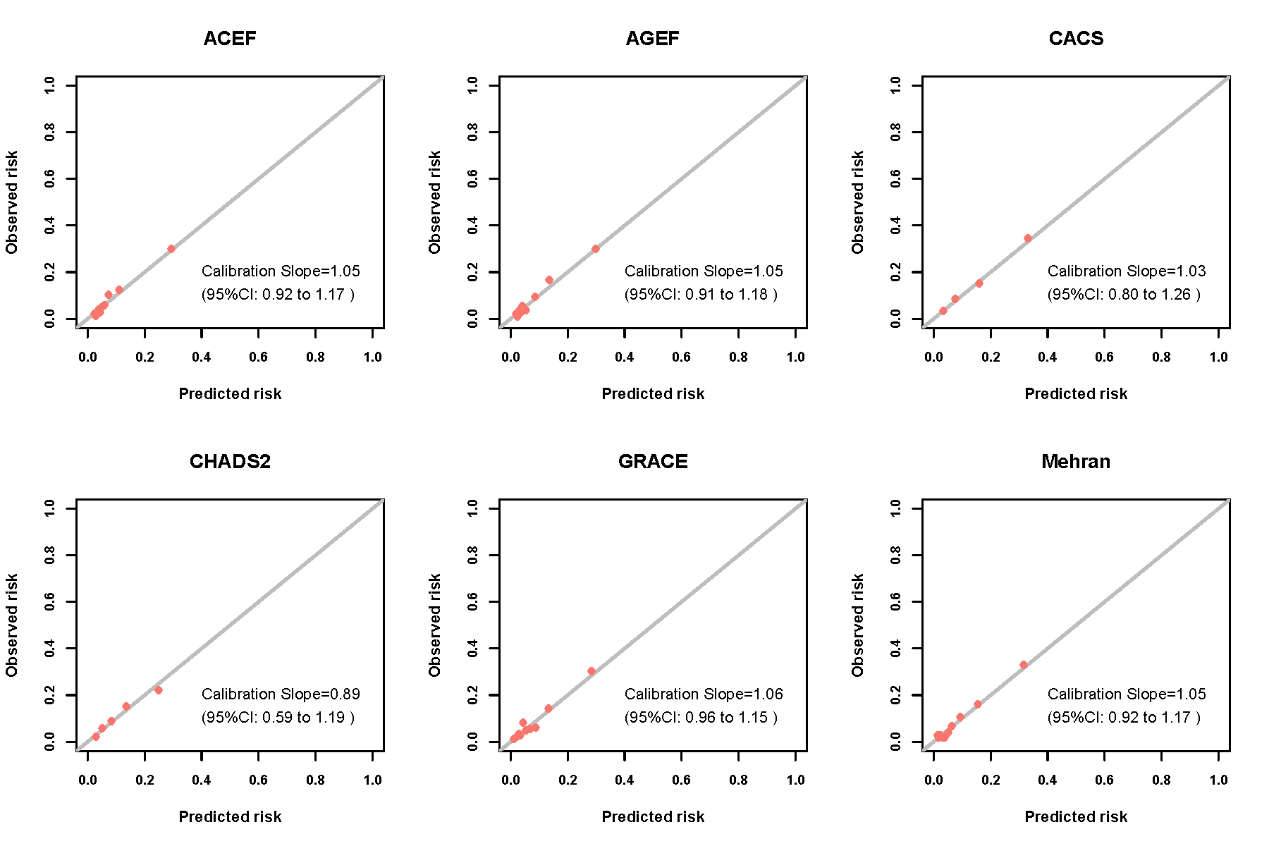


*ACEF, age, creatinine, and ejection fraction; AGEF, age, glomerular filtration rate, and ejection fraction; CACS, Canada acute coronary syndrome; GRACE, global registry for acute coronary events; CI, confidence interval.*

**Figure S4.** Decision curves of the risk scores for major adverse clinical events.


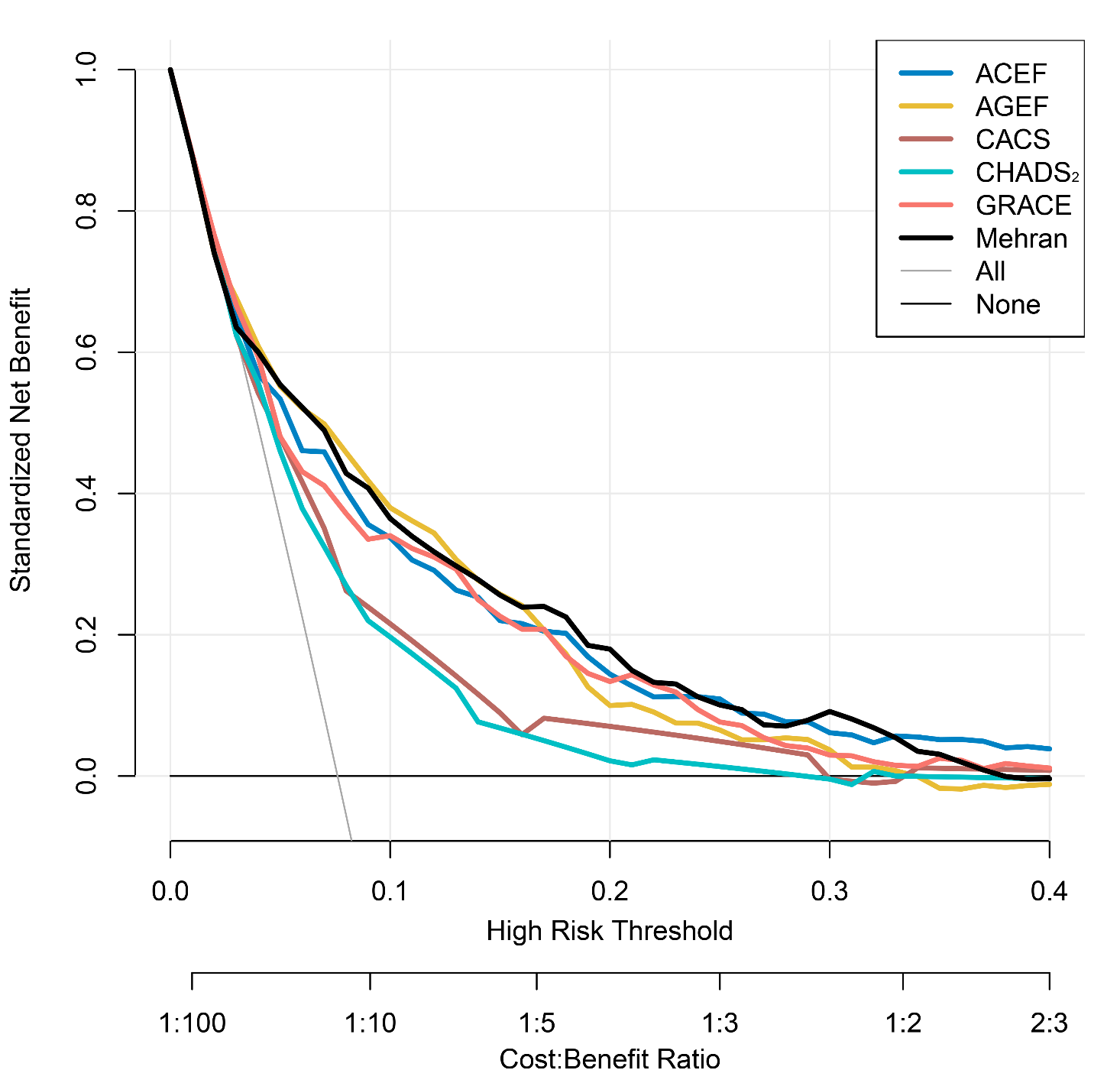


*ACEF, age, creatinine, and ejection fraction; AGEF, age, glomerular filtration rate, and ejection fraction; CACS, Canada acute coronary syndrome; GRACE, global registry for acute coronary events.*

**Table S1. Variables in risk scores evaluated.**

**Risk Scores Variables Score**

**ACEF score**

Age/Left ventricular ejection fraction (%) +1, if serum creatinine > 2.0 mg/dL

**AGEF score**

Age/Left ventricular ejection fraction (%) +1, if Estimated glomerular filtration rate <

60mL/min/1.73 m^2^

**CACS score**

Age ≥75 years 1

Killip > I 1

Systolic blood pressure <100 mm Hg 1

Heart rate >100 beats/min 1

**CHADS_2_ score**

Congestive heart failure 1

Hypertension 1

Age ≥ 75 years 1

Diabetes mellitus 1

Previous stroke/ transient ischemic attack 2

**GRACE score**

***Age (years)***

< 30 0

30-39 8

40-49 25

50-59 41

60-69 58

70-79 75

80-89 91

≥90 100

***Heart rate (beats/min)***

<50 0

50-69 3

70-89 9

90-109 15

110-149 24

150-199 38

>200 46

***Systolic blood pressure(mmHg)***

<80 58

80-99 53

100-119 43

120-139 34

140-159 24

160-199 10

>200 0

***Creatinine(mg/dL)***

0-0.39 1

0.4-0.79 4

0.8-1.19 7

1.2-1.59 10

1.6-1.99 13

2-3.99 21

>4 28

***Killip’s classification***

I 0

II 20

III 39

IV 59

***Cardiac arrest at admission***  39

***Increased cardiac markers***  14

***ST-Segment deviation*** 28

**Mehran score**

Age >75 years 4

Anemia* 3

Diabetes mellitus 3

Chronic heart failure 5

Intra-aortic balloon pump 5

Hypotension 5

Contrast media volume 1

Serum creatinine >1.5 mg/dL 4

* Anemia defined as baseline hematocrit 39% for men and 36% for women.

*ACEF: age, creatinine, and ejection fraction; AGEF: age, glomerular filtration rate, and ejection fraction; CACS: Canada acute coronary syndrome; GRACE: global registry for acute coronary events.*

**Table S2.** Predictive accuracy of the risk scores for infection in patients with different WBC count.

| **Events** | **ACEF** | **AGEF** | **CACS** | **CHADS_2_** | **GRACE** | **Mehran** |
| --- | --- | --- | --- | --- | --- | --- |
|  | **AUC (95%CI) P1/P2** | | | | | |
| **WBC<10**  **×10^9^/L** | 0.775  (0.719-0.832) | 0.777  (0.720-0.833) | 0.722  (0.665-0.778) | 0.671  (0.608-0.735) | 0.772  (0.717-0.827) | 0.751  (0.688-0.815) |
|  | Reference | Reference | 0.108/0.092 | 0.002/0.002 | 0.902/0.861 | 0.433/0.385 |
| **WBC≥10**  **×10^9^/L** | 0.750  (0.717-0.783) | 0.775  (0.743-0.807) | 0.757  (0.727-0.787) | 0.703  (0.669-0.737) | 0.803  (0.775-0.832) | 0.769  (0.737-0.802) |
|  | Reference | Reference | 0.700/0.304 | 0.015/<0.001 | 0.001/0.050 | 0.272/0.718 |

P1: other risk scores (but not AGEF) versus ACEF; P2: other risk scores (but not ACEF) versus AGEF.

*WBC, white blood cell; ACEF, age, creatinine, and ejection fraction; AGEF, age, glomerular filtration rate, and ejection fraction; CACS, Canada acute coronary syndrome; GRACE, global registry for acute coronary events; AUC, area under the curve; CI, confidence interval.*

**Table S3.** Predictive accuracy of the risk scores for infection in male and female.

| **Events** | **ACEF** | **AGEF** | **CACS** | **CHADS_2_** | **GRACE** | **Mehran** |
| --- | --- | --- | --- | --- | --- | --- |
|  | **AUC (95%CI) P1/P2** | | | | | |
| **Male** | 0.758  (0.727-0.790) | 0.772  (0.741-0.803) | 0.761  (0.731-0.790) | 0.684  (0.651-0.718) | 0.798  (0.770-0.827) | 0.758  (0.725-0.791) |
|  | Reference | Reference | 0.907/0.507 | <0.001/<0.001 | 0.007/0.063 | 0.973/0.356 |
| **Female** | 0.699  (0.630-0.768) | 0.757  (0.694-0.821) | 0.680  (0.619-0.742) | 0.669  (0.603-0.736) | 0.759  (0.697-0.821) | 0.730  (0.661-0.798) |
|  | Reference | Reference | 0.644/0.037 | 0.499/0.021、 | 0.105/0.957 | 0.470/0.476 |

P1: other risk scores (but not AGEF) versus ACEF; P2: other risk scores (but not ACEF) versus AGEF.

*ACEF, age, creatinine, and ejection fraction; AGEF, age, glomerular filtration rate, and ejection fraction; CACS, Canada acute coronary syndrome; GRACE, global registry for acute coronary events; AUC, area under the curve; CI, confidence interval.*

**Table S4.** Predictive accuracy of the risk scores for infection in patients with or without hypertension.

| **Events** | **ACEF** | **AGEF** | **CACS** | **CHADS_2_** | **GRACE** | **Mehran** |
| --- | --- | --- | --- | --- | --- | --- |
|  | **AUC (95%CI) P1/P2** | | | | | |
| **With hypertension** | 0.752  (0.715-0.789) | 0.792  (0.757-0.826) | 0.743  (0.707-0.778) | 0.709  (0.670-0.747) | 0.795  (0.761-0.829) | 0.798  (0.760-0.835) |
|  | Reference | Reference | 0.669/0.015 | 0.055/<0.001 | 0.021/0.845 | 0.025/0.752 |
| **Without hypertension** | 0.739  (0.694-0.784) | 0.742  (0.697-0.787) | 0.747  (0.707-0.787) | 0.690  (0.647-0.733) | 0.783  (0.743-0.823) | 0.760  (0.715-0.805) |
|  | Reference | Reference | 0.736/0.837 | 0.051/0.034 | 0.030/0.040 | 0.388/0.447 |

P1: other risk scores (but not AGEF) versus ACEF; P2: other risk scores (but not ACEF) versus AGEF.

*ACEF, age, creatinine, and ejection fraction; AGEF, age, glomerular filtration rate, and ejection fraction; CACS, Canada acute coronary syndrome; GRACE, global registry for acute coronary events; AUC, area under the curve; CI, confidence interval.*
